# Designing Protective Systems and Selecting Materials for Motorsport Safety Through Biomechanical Evidence: A Scoping Review

**DOI:** 10.3390/bioengineering13101159

**Published:** 2026-10-03

**Authors:** Jiongxiang Zhao, Enze Shao, Zilong Lu, Jun Wen, Chao Zeng, Tibor J. Goda, Viktória Tafferner-Gulyás, Dong Sun, Yaodong Gu

**Affiliations:** 1 Research Academy of Medicine Combining Sports, Ningbo No. 2 Hospital, Ningbo 315010, China; zhaojiongxiang@outlook.com (J.Z.); enze.shao@phd.uni-obuda.hu (E.S.); zilonglu2002@outlook.com (Z.L.); wennjunn666@gmail.com (J.W.); 2 Faculty of Sports Science, Ningbo University, Ningbo 315211, China; 3 ANTA (China) Co., Ltd., Xiamen 316008, China; 4 Research Center for Molecular Exercise Science, Hungarian University of Sports Science, 1123 Budapest, Hungary; 5 Doctoral School on Safety and Security Sciences, Óbuda University, 1088 Budapest, Hungary; goda.tibor@bgk.uni-obuda.hu; 6 Doctoral School of Materials Sciences and Technologies, Óbuda University, 1034 Budapest, Hungary; zengchao283@gmail.com; 7 Institute of Technical Physics and Materials Science, HUN-REN Centre for Energy Research, 1121 Budapest, Hungary; 8 Institute for Natural Sciences and Basic Subjects, Bánki Donát Faculty of Mechanical and Safety Engineering, Óbuda University, 1088 Budapest, Hungary; 9 John von Neumann Faculty of Informatics, Óbuda University, 1034 Budapest, Hungary; tafferner.viktoria@uni-obuda.hu

**Keywords:** biomechanics, motorsport safety, finite element analysis, protective systems, injury prevention

## Abstract

Motorsport remains high risk despite advances in crash protection, vehicle design, and medical response. Effective injury prevention requires understanding interactions among crash loading, human injury tolerance, protective-system design, and material behavior. This scoping review searched PubMed, Web of Science, IEEE Xplore, and Scopus for English-language studies from 2000 to July 2026 addressing motorsport safety, injury biomechanics, finite element analysis or modeling, protective-system design, or impact-mitigation materials. Study characteristics were charted, and evidence was narratively synthesized following scoping-review principles. Fifty-six studies were included. Injury prevention increasingly draws on head-kinematic field measurements, injury epidemiology, region-specific finite element analyses, and studies of helmets, restraints, seats, and energy-absorbing structures. The most direct evidence concerns integrated head-neck protection, restraint and seat geometry, and the use of finite-element methods to interpret local injury mechanisms. Emerging material strategies are promising but rely mainly on component-level or simulation-based evidence rather than driver-level injury outcomes. Overall, biomechanics, protective-system design, and material selection are increasingly integrated, but evidence remains uneven. Real-world injury surveillance is limited, simulation studies predominate over field outcomes, and direct evidence linking emerging materials to driver-level injury reduction remains sparse. Progress requires better surveillance datasets, stronger motorsport-specific validation, and more driver-centered protective design.

## 1. Introduction

Motorsport remains a high-risk sport despite major improvements in vehicle structure, restraints, helmets, and medical response [1]. Ongoing technological innovations in motorsport have significantly augmented the performance capabilities of drivers and vehicles, the sport remains inherently hazardous. Participants are continuously exposed to a high-risk environment, distinguished by elevated probabilities of severe injuries and fatal outcomes [2]. This paradoxical relationship between technological progress and persistent safety challenges underscores the complex nature of motorsport risk management. Additionally, existing literature indicates that drivers face life-threatening risks not only from non-impact injuries (e.g., extreme G-forces, hyperthermia, and cardiovascular strain) [3], but also from motorsport crashes [4,5].

Motorsport crash events represent complex biomechanical phenomena characterized by multiple interacting variables. Researchers require systematic analysis of both crash mechanisms and environmental conditions [6]. As an effective approach for evaluating protective equipment, several injury standards have been developed from biomechanical data. These injury criteria typically correlate engineering measurements or stimuli such as acceleration [7], force [8], or deflection, to a specific injury mechanism. For instance, J. Patalak et al. conducted research investigating the relationship between initial driver positioning, seat ramp design parameters, and thoracolumbar loading during frontal impact scenarios [9]. To validate their motorsport restraint system model, the researchers used finite element modeling (FEM) with the industry-standard 50th percentile male Hybrid III FEM, establishing a measurable link between seat design variables and injury risk metrics. Therefore, biomechanical assessment methodologies offer effective frameworks for analyzing driver performance metrics and injury mechanisms in motorsport applications [10]. A compelling example of this is the Head and Neck Support (HANS) device, which was developed by Dr. Robert Hubbard and colleagues [11]. Regarding other cases, biomechanical tools support the development and evaluation of a range of protective equipment for drivers, such as safety harnesses, head and neck restraints, full-face helmets, and energy-absorbing toe-board foam [12]. In the future, biomechanics tools will have great potential to explain the effectiveness and protective mechanisms of new safety equipment.

Distinct materials used in protective equipment provide varying levels of performance for drivers [13]. In the case of helmets, the driver’s helmet, which was initially made of fabric or leather, had developed into metal helmets by the 1950s. Although metal helmets offered improved protection against blunt force impacts, the metal helmet may cause extra lethal flexion-distraction forces at the craniovertebral junction [14], if the mass exceeded 1.5 kg. Consequently, the search for lightweight materials with enhanced energy-absorbing properties is a key focus in the development of safer motorsport helmets. JC Najmon et al. proposed that a bio-inspired and compliant-mechanism lattice could replace the conventional foamed polypropylene liner in motorsport helmets [15]. By computer simulation technology, this composite material was evaluated and demonstrated to deliver improved performance in energy absorption and impact mitigation. Apparently, advances in material science continue to enhance driver safety by enabling the development of innovative protective solutions.

Although previous studies have examined individual aspects of motorsport safety, a focused synthesis linking injury biomechanics to both protective-system design and material selection is still lacking. This scoping review therefore examines what evidence is available, how that evidence has informed the design and evaluation of protective systems, and where the main translational gaps remain between biomechanical knowledge and practical driver protection.

## 2. Materials and Methods

### 2.1. Information Sources and Search Strategy

This study was conducted as a scoping review because the literature relevant to motorsport safety spans epidemiology, field biomechanics, laboratory testing, finite element analysis (FEA), and engineering design. A scoping approach was therefore more suitable than pooled effect estimation or narrow intervention comparison. The review process was structured to make study identification, selection, and evidence charting transparent and was reported with reference to PRISMA-ScR principles. No protocol for this scoping review was prospectively registered in PROSPERO or another public repository.

A structured literature search was performed in PubMed, Web of Science, IEEE Xplore, and Scopus to identify English-language studies published between January 2000 and July 2026. The searches were conducted in July 2026. The exact calendar dates of the individual database searches were not recorded. The search strategy was adapted to each database and centered on motorsport-related terms, including motorsport, automotive racing, auto racing, racecar, competitive motor vehicle racing, and high-performance vehicle racing. Retrieved records were subsequently screened for relevance to injury biomechanics, finite-element analysis, protective systems, and impact-mitigation materials. The complete database-specific search strategies, search limits, search dates, and numbers of records identified are provided in Table 1.

Reference lists of eligible studies were also screened manually to identify additional records. For each included study, the review charted publication type, anatomical focus, motorsport context or adjacent application domain, protective system or material category, study design, and principal biomechanical or engineering finding.

### 2.2. Eligibility Criteria

Studies were included for review only if they met all of the following criteria: (1) only original research articles published in English-language journals were included; (2) studies reported original or technically substantive evidence in the form of peer-reviewed journal articles or authoritative technical papers; (3) included articles addressed motorsport safety, injury biomechanics, protective-system design, finite element or computational injury analysis, or materials relevant to impact mitigation; (4) included studies contained sufficient methodological or technical detail to support data extraction and qualitative synthesis. Studies from adjacent automotive biomechanics research were retained only when they were directly relevant to motorsport injury mechanisms, occupant loading, restraint performance, or protective design.

For studies conducted outside motorsport, direct relevance was judged according to whether the investigated loading condition, occupant posture, restraint configuration, injury mechanism, or protective technology could reasonably be transferred to a motorsport driver-protection context. Studies were retained when they addressed at least one of the following: (i) impact directions or crash pulses relevant to motorsport; (ii) driver seating, harness, helmet, head-and-neck restraint, or cockpit geometry; (iii) injury mechanisms involving the head, neck, thorax, spine, pelvis, or lower extremities that are relevant to racing crashes; or (iv) materials, structures, or energy-absorbing concepts intended for occupant protection or impact mitigation. Studies focused exclusively on vehicle-level crashworthiness without a clear connection to occupant protection were excluded.

### 2.3. Study Selection

Two authors (JZ and ES) independently screened the retrieved records in a staged process involving manual duplicate removal, title and abstract screening, and full-text eligibility assessment. Duplicate records were identified by comparing titles, authors, publication years, journals, and DOIs where available. A total of 890 duplicate records were removed manually, leaving 760 records for title, abstract, and keyword screening. Of these, 440 records were excluded because they were unrelated to motorsport or automotive racing. The remaining 320 reports were sought for retrieval and assessed for eligibility at the full-text stage. A further 264 reports were excluded because they were not related to the review scope, were not published in English, were review articles or conference abstracts, or did not provide sufficient information on methods or results. The final inclusion set comprised 56 studies. Disagreements regarding study inclusion were resolved through discussion with the senior authors (TG and YG) until consensus was reached. No automated deduplication software or algorithm was used. All duplicate records were identified and removed manually by comparing titles, authors, publication years, journals, and DOIs where available.

### 2.4. Evidence Synthesis

Data were charted from each included study using a structured extraction framework. One reviewer conducted the data charting, and a second reviewer independently checked the completed chart for all 56 included studies. The extracted variables included bibliographic information, study design, motorsport series or adjacent application context, participant or model characteristics, crash or loading conditions, restraint or protective intervention, material or structural configuration, biomechanical variables, injury or performance outcomes, validation approach, principal findings, and reported limitations. Any discrepancies or ambiguities were resolved through discussion and, when necessary, consultation with the senior authors. The completed study-level extraction is provided in Supplementary Table S1. The extraction also recorded the qualitative evidence maturity of each study and whether a formal Technology Readiness Level (TRL) was explicitly reported by the original study.

These variables were used to classify the included evidence into four thematic domains: (1) injury mechanisms and biomechanical evidence, (2) finite-element analysis and computational modeling, (3) protective-system design and performance evaluation, and (4) material selection and impact mitigation. Because the included studies differed substantially in design, study object or model, loading condition, outcome definition, and validation procedure, a quantitative meta-analysis was not performed. Instead, the findings were synthesized narratively and summarized within these thematic domains.

In addition to describing study characteristics and findings, the synthesis considered the directness, methodological scope, validation status, outcome relevance, and transferability of each evidence type. Direct motorsport field studies were considered most informative for describing real-world exposure and crash loading, whereas laboratory experiments and computational models were interpreted primarily in relation to mechanism identification and design evaluation. Materials studies were further considered according to whether they provided material-level or component-level performance data and whether these data were linked to driver-level injury mitigation. These considerations were used to distinguish relatively established engineering evidence from emerging or preliminary concepts.

Formal risk-of-bias assessment was not undertaken because the primary aim of this scoping review was to map a heterogeneous multidisciplinary evidence base rather than estimate pooled intervention effects. Nevertheless, the included studies were not treated as equivalent. Differences in study design, motorsport specificity, sample or model characteristics, loading conditions, validation procedures, outcome measures, and reported limitations were considered when interpreting the strength and practical relevance of the findings.

## 3. Results

### 3.1. Search Result

**Figure 1:**
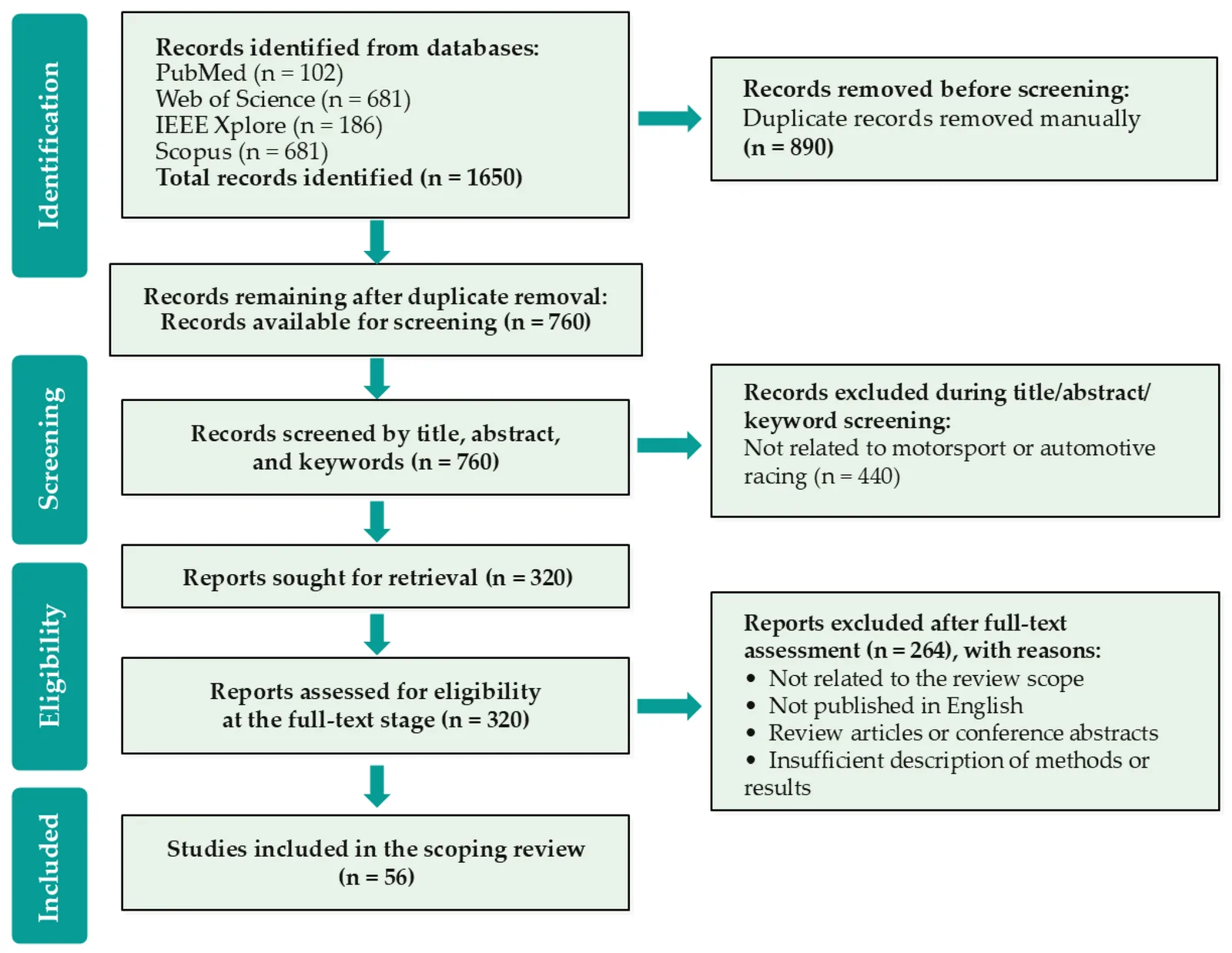
PRISMA-ScR flow diagram of the literature search and study selection.

Figure 1 summarizes the literature selection process. The database search identified 1650 records, including 102 from PubMed, 681 from Web of Science, 186 from IEEE Xplore, and 681 from Scopus. Manual removal of 890 duplicate records left 760 records for title, abstract, and keyword screening. During this stage, 440 records were excluded as unrelated to motorsport or automotive racing. The remaining 320 reports underwent full-text eligibility assessment. Of these, 264 reports were excluded because they did not meet the predefined eligibility criteria, while 56 studies were included in the final qualitative synthesis.

The 56 included studies were organized into four primary evidence domains: injury mechanisms and biomechanical evidence, finite-element analysis and computational modeling, protective-system design, and material selection and impact mitigation (Table 2). Data were charted from all 56 included studies using a standardized extraction framework. The extracted variables included study design, motorsport or adjacent context, sample or model characteristics, loading or test conditions, protective system or material, outcome measures, validation method, principal findings, study limitations, evidence maturity, and whether a formal Technology Readiness Level (TRL) was reported by the original study. The complete data-charting table is provided in Supplementary Table S1.

The 56 included studies formed a methodologically heterogeneous evidence corpus, comprising observational and epidemiological investigations, biomechanical experiments, finite-element and computational modeling, protective-system evaluations, and materials or component-testing studies. To provide a quantitative overview of this corpus, the studies were categorized according to their primary study design, publication period, and main biomechanical or protection focus (Table 3). Each study was assigned to one primary category within each classification dimension, while detailed study-level characteristics are provided in Supplementary Table S1. This classification supported the thematic synthesis of injury biomechanics, injury prediction, protective-system design, and material-selection strategies in motorsport safety.

Computational and numerical studies represented the largest methodological group, accounting for 23 of the 56 included studies, while 22 studies were published between 2020 and 2026.

### 3.2. Injury Mechanisms and Biomechanical Insights in Motorsport Accidents

#### 3.2.1. Key Biomechanical Studies

Motorsport exposes drivers to a broad spectrum of injury mechanisms, with the head, neck, spine, and extremities representing the most consistently affected regions in the available literature [18]. Epidemiological studies indicate that although many injuries are musculoskeletal and non-fatal, head and cervical injuries remain of particular concern because of their severity and their relevance to long-term neurological and spinal outcomes [3,18]. This part of the literature provides the basic injury pattern, but more direct insight into crash loading comes from field-based head-kinematics studies and related biomechanical measurements. Injury severity was interpreted using the Abbreviated Injury Scale (AIS), a standardized anatomical injury-severity classification system. Representative injury characteristics and biomechanical indicators reported in the included studies are summarized in Table 4.

Direct measurement studies provide some of the clearest motorsport-specific evidence. Early work on in-race accelerometer systems established that head acceleration and motion could be measured reliably in high-impact racing environments. More recent instrumented mouthpiece studies in grassroots dirt-track racing showed that head loading is substantially greater during crashes than during routine laps, with crash-related peak linear acceleration reaching a median of 13.4 g and a 95th percentile above 36 g [7,16]. Pilot head-kinematics studies in stock-car racing extended these findings by showing that on-board and driver-based sensing can provide usable race-environment data for evaluating restraint performance and interpreting crash severity in relation to driver exposure [24].

Field and crash-based investigations further suggest that chassis-level loading and impact sequence are meaningful predictors of injury risk. Weaver et al. reported that crashes involving peak vehicle accelerations of at least 50 g were associated with substantially greater head-injury risk than lower-severity crashes [26]. In a complementary injury-risk analysis, Patalak et al. showed that larger changes in velocity, higher peak acceleration, and a greater number of impacts were all associated with higher injury probability, with multivariable models showing good discriminatory ability [12]. Together, these findings indicate that motorsport injury risk is influenced not only by absolute peak loading but also by impact direction, repeated loading, and crash complexity.

Motorsport-specific injury-threshold data remain limited, so related automotive biomechanics studies are still used as reference points for interpreting injury tolerance. Head and neck injury assessment reference values derived from cadaveric, animal, and crash-test data remain useful for contextualizing measured accelerations and restraint-system loading [19]. These translated benchmarks should be interpreted with caution, however, because seating geometry, restraint architecture, crash direction, and driver posture differ from those in conventional road-vehicle scenarios.

Taken together, the available literature supports three consistent points. Direct head-kinematics monitoring in motorsport now provides meaningful field-based biomechanical data, especially in grassroots and stock-car settings [7,16,24]. Injury risk depends on both the magnitude and the pattern of loading [27], including rotational motion, repeated impacts, and crash direction [12,26,44]. At the same time, the evidence base remains limited by small cohorts, uneven instrumentation across racing categories, and the continued lack of standardized real-world injury surveillance data. The injury-mechanism evidence was most direct when real-world crash data or measured driver head kinematics were available. These studies provided motorsport-specific information that cannot be fully obtained from laboratory surrogates. However, their generalizability was limited by small cohorts, differences in instrumentation, incomplete crash capture, and uneven medical outcome recording across racing categories. Epidemiological studies were useful for identifying injury patterns and risk factors, but generally provided less detail about the mechanical pathways that produced individual injuries. Field measurements and epidemiological findings should therefore be interpreted as complementary rather than interchangeable forms of evidence.

#### 3.2.2. Injury Prediction via FEA

FEA has become one of the most useful tools for investigating motorsport injury mechanisms because it allows detailed simulation of driver-vehicle interactions under crash loading. In the reviewed literature, finite element models were used to predict tissue deformation, identify region-specific injury mechanisms, and examine how seating, restraint geometry, and cockpit design influence injury risk [17,29,35].

The underlying models ranged from whole-body human body models to region-specific representations of the cervical spine, thorax, pelvis, and lower extremities [31,32,36,37]. Many of these models originated in automotive safety research and were later translated into motorsport-relevant interpretation, whereas others were applied more directly to race-car conditions such as National Association for Stock Car Auto Racing and endurance-racing frontal impacts [25,29]. This distinction matters because motorsport-specific conclusions are strongest when crash boundary conditions, posture, and restraint geometry are represented explicitly rather than inferred from adjacent automotive settings.

As summarized in Table 5, FEA consistently identified the thoracolumbar spine as a major site of injury risk in frontal and frontal-oblique impacts. Compression, wedge, and burst fracture patterns were associated with axial loading transmitted through the pelvis and seat, with seat ramp angle, driver posture, and restraint geometry all affecting local loading [9,23,25]. Thoracic injuries, including rib and sternum fractures, were linked to steering-wheel contact or concentrated restraint loading during high deceleration [29,35]. Head and neck loading also increased in severe frontal and lateral conditions, while pelvic and lower-extremity models clarified injury pathways related to side intrusion, knee-thigh-hip loading, and acetabular tolerance [30,32,34].

FEA has also been used to examine design countermeasures. Motorsport-relevant studies indicate that changes in seat angle, padding, and belt anchorage can reduce spinal loading, while simplified soft-body or reduced-order models may offer faster alternatives for some crashworthiness problems [25,39]. Region-specific simulations have also improved understanding of cervical loading, dynamic foraminal narrowing, and ligament strain, which are difficult to measure directly in physical tests [20,22,48]. Related neck studies have also pointed to vascular deformation mechanisms, including dynamic vertebral artery elongation during frontal and side impact loading, which further illustrates the range of cervical responses that may not be apparent from gross kinematics alone [21,42]. The computational evidence provided detailed information about local tissue loading and the effects of posture, seat geometry, restraint configuration, and impact direction. Its main strength was the ability to examine variables that are difficult to measure directly in real crashes. However, the validity of these conclusions depended on the human body model, material properties, boundary conditions, crash pulse, and validation data. Models developed in adjacent automotive contexts were useful for hypothesis generation and comparative design analysis, but their results should not be interpreted as fully motorsport-specific unless racing-relevant posture, restraint geometry, and loading conditions were explicitly represented. The current evidence therefore supports FEA as a mechanism and design tool, while providing more limited evidence for direct prediction of real-world driver injury outcomes.

### 3.3. Protective-System Design and Performance Evaluation

#### 3.3.1. Helmet Design and Head-Neck Protection

Motorsport helmet design has shifted from isolated impact protection toward integrated head-neck protection guided by crash investigation, biomechanical testing, and computational modeling (Table 6). Modern helmet systems combine high-strength shells, energy-absorbing liners, and compatibility with head-and-neck restraints to manage both head injury risk and cervical loading.

Current studies no longer treat the helmet as a stand-alone component. Its performance is evaluated in relation to restraint systems, seat geometry, and driver anthropometry [28,40]. This system-level view is one reason helmet development has become closely tied to broader cockpit protection strategies.

A major development in this area was the adoption of the HANS device and related restraint concepts. Experimental and computational studies indicate that these systems can reduce upper-neck tension, shear force, and extension moment during severe frontal impacts, providing a biomechanical rationale for their protective role [41,43,48]. At the same time, helmet design has moved toward composite shells, improved energy-absorbing liners, and better system compatibility with harnesses and tethers [28,40,49].

Recent simulation studies further indicate that helmet mass, driver body size, and restraint integration can influence head and neck injury metrics, whereas external accessories such as visors or cameras appear to have smaller effects under modeled conditions [28,29]. Overall, the more direct evidence in this section concerns integrated helmet–restraint systems evaluated through biomechanical testing or computational analysis, whereas sensor-enabled and highly individualized helmet concepts remain at an earlier stage of development [49].

#### 3.3.2. Body Protection and Seat Design

For body protection, the evidence is stronger for seating and restraint geometry than for textile mechanics alone. Racing suits remain essential for thermal protection, and aramid-based fabrics such as Nomex are widely used because they combine fire resistance with acceptable mobility. One study also identified a design limitation for female drivers: unisex suit patterns may fit poorly at the shoulder, waist, hip, and crotch [46].

Seat-related evidence is more directly connected to biomechanical injury mitigation. Experimental and computational studies show that seat configuration, ramp angle, foam support, and restraint integration can materially influence thoracolumbar loading during frontal impacts [8,9,47]. The clearest practical implication is that seat support and restraint geometry affect injury mitigation more consistently than apparel changes alone, even though both matter for the overall driver-protection system.

Compared with apparel-focused studies, seat and restraint studies provide a clearer biomechanical link to driver loading; however, most outcomes were surrogate biomechanical measures rather than clinically confirmed injuries.

#### 3.3.3. Spine Protection Systems

Spine protection in motorsport depends on both cervical restraint and thoracolumbar load management. The strongest evidence concerns HANS and related head-neck restraint systems, which reduce upper-neck tension, shear, and extension during severe frontal impacts when properly integrated with the helmet, harness, and seat [43,48,52,58].

Below the cervical region, FE-based studies show that thoracolumbar injury risk is strongly influenced by seat ramp angle, posture, and restraint geometry [9,23,25]. Increased axial loading through the pelvis-seat path is associated with compression- and burst-type injuries, whereas better load distribution can reduce local spinal demand. The available evidence therefore suggests that spine protection is best understood as a cockpit-system problem rather than the performance of a single device.

However, the available studies primarily evaluated loading variables, injury metrics, or model responses, and relatively few linked these outcomes to confirmed driver injuries in real-world racing. This limits the extent to which laboratory or computational improvements can be interpreted as demonstrated reductions in clinical injury risk.

### 3.4. Material Selection for Motorsport Safety

In motorsport safety, material selection should be evaluated according to the protective task, loading condition, deformation behavior, durability, manufacturability, and certification feasibility rather than by strength-to-weight ratio or energy absorption alone. The included studies differed substantially in their level of validation. Some investigated established materials in component-level or crash-related tests, whereas others evaluated emerging concepts mainly through material characterization, simulation, or small-scale laboratory experiments. These differences are important when judging the practical readiness of materials for driver protection.

#### 3.4.1. High-Performance Materials

For clarity, the material evidence included both driver-protective components, such as cockpit padding, toe-board foam, seat materials, and helmet liners, and vehicle-level crash structures, such as impact attenuators and sacrificial structures. Vehicle-level materials were retained only when their energy-absorption function was relevant to driver protection; however, their findings should not be interpreted as direct evidence of driver-level injury reduction.

Material selection in motorsport safety requires trade-offs among low mass, structural efficiency, manufacturability, and crash-energy management. This line of work also extends to certified composite energy absorbers, where thin-walled structures are designed not only for high specific energy absorption but also for predictable collapse behavior under regulated crash conditions [53]. Carbon-fiber-reinforced polymers (CFRPs) remain central to this work, because they provide high specific strength and stiffness while still allowing progressive energy absorption in crashworthy structures such as thin-walled impact components [62].

Hybrid composite systems may improve manufacturability and reduce production cost relative to pure CFRPs, although these gains can be offset by greater susceptibility to delamination and machining-related surface damage [55]. At the component level, specialized polymers and energy-absorbing foams also remain important in cockpit protection. Toe-board energy-absorbing materials, for example, have been shown to reduce tibial axial load and foot acceleration under lower-extremity impact conditions [50,51]. Formula Society of Automotive Engineers studies have also shown that impact attenuator performance can be improved through geometry-driven optimization, with material selection and wall-thickness distribution both affecting peak force and crash-force efficiency [60,61]. Collectively, these findings suggest that high-performance materials should be selected according to component-level loading paths and deformation requirements rather than by strength-to-weight ratio alone. However, material-level or component-level improvements cannot automatically be interpreted as evidence of reduced driver injury risk unless they are evaluated within a representative protective system or crash configuration.

#### 3.4.2. Innovative Materials and Emerging Technologies

Recent motorsport safety research has expanded beyond conventional composites to include sandwich structures, metamaterials, additive manufacturing, and bio-inspired energy-absorbing concepts. These approaches are attractive because they aim to improve crash performance while also addressing manufacturability, weight reduction, and, in some cases, sustainability. At present, however, most of the evidence in this area remains at the level of structural performance or design feasibility rather than direct driver-level injury reduction.

Hybrid aluminum-honeycomb sandwich systems remain relevant for safety-critical structures because they combine stiff face sheets with an energy-absorbing core, allowing efficient crash-energy management despite complex local failure modes [56]. Related experimental work on sandwich beams with CFRP face sheets and 3D-printed cores suggests that core architecture can materially influence stiffness-to-weight and strength-to-weight performance, which is directly relevant to lightweight safety structures in motorsport [54]. Even so, the evidence is still weighted toward engineering performance, and direct links to driver-level injury reduction remain limited [13].

Additive manufacturing and topology optimization are also enabling more complex, weight-efficient geometries for safety components [59]. Studies of natural-fiber composites, recycled composites, and auxetic or cellular foams point to possible alternatives for selected energy-absorbing applications [15,57,63,64]. Their relevance to motorsport safety will depend on validation under realistic loading, packaging, and certification constraints.

Evidence for auxetic foams, cellular lattices, metamaterials, natural-fiber composites, recycled composites, and additively manufactured structures was comparatively less mature. Most studies reported simulation, material-level, or component-level outcomes, while few evaluated durability, environmental conditioning, manufacturing variability, integration with existing protective systems, or driver-level injury outcomes. These concepts should therefore be interpreted as promising design strategies rather than validated replacements for established protective materials.

The maturity of the material evidence varied across the included studies. Conventional composite structures, honeycomb systems, and polymeric energy absorbers had relatively more developed component-level or crash-related evidence, whereas auxetic, cellular, metamaterial, natural-fiber, recycled, and additively manufactured concepts were supported mainly by simulation or laboratory-scale testing. These differences are summarized according to the reported application, validation level, and translational status in Table 7.

Overall, the reviewed materials showed a gap between engineering feasibility and demonstrated driver-protection effectiveness. Established materials had more evidence from component or crash-related testing, but their performance remained dependent on geometry, manufacturing quality, loading direction, and environmental conditions. Emerging material concepts offered promising opportunities for weight reduction and tunable energy absorption, but their practical adoption will require motorsport-relevant impact validation, durability testing, manufacturing repeatability, system integration, and certification evidence.

## 4. Discussion

This scoping review identified clear differences in the directness and maturity of the available evidence. Real-world head-kinematics studies and motorsport-specific investigations provided the most direct information about driver exposure and crash loading. Finite-element studies contributed detailed information about local injury mechanisms and allowed the effects of posture, restraint configuration, and seat geometry to be examined, but their conclusions depended on model validation and the representativeness of the simulated loading conditions. Protective-system studies were most informative when they evaluated integrated helmet, restraint, and seat configurations, whereas many emerging material concepts were supported mainly by material-level testing or computational analysis rather than driver-level injury outcomes.

### 4.1. Challenges in Converting Between Research and Practice

One of the main barriers is that much of the available evidence is generated under laboratory or simulation conditions rather than through standardized real-world crash surveillance. Although FEA and related modeling approaches are essential for mechanism analysis and design optimization, their practical value still depends on validation against representative crash conditions and implementable engineering constraints [29,62]. This gap is especially important in grassroots and amateur motorsport, where instrumentation, medical surveillance, and access to advanced safety systems remain more limited than in elite series [3,44].

Regulatory implementation also tends to lag behind engineering development. New devices and materials must satisfy certification, manufacturing, and compatibility requirements before widespread adoption, which can slow the translation of concepts such as metamaterials, advanced liners, and novel crash structures [13,53,62]. Inconsistent data collection and limited data sharing also make it difficult to compare findings across series and to build harmonized rule sets [7,44].

A practical translational pathway in motorsport safety involves several linked stages. A biomechanical or epidemiological finding must first be converted into a measurable design target, such as a limit for head acceleration, neck force and moment, thoracic loading, spinal compression, or lower-extremity force. The proposed protective system must then be evaluated using representative sled, drop, component, or crash tests, supported by validated computational models where appropriate. To inform helmet standards such as the FIA 8860 series or applicable seat-homologation requirements, the performance measure must be reproducible, associated with a defined loading condition, and compatible with manufacturing, packaging, fit, and durability requirements. Only after these steps can evidence contribute to technical specifications, homologation procedures, or competition regulations. The HANS device provides an illustrative example of this type of translation from a biomechanical understanding of head–neck loading to an engineered and regulated protective system. However, the present review does not establish that any single included study directly caused a specific regulatory change. For small-scale innovations, the cost, duration, and technical burden of certification testing may further delay adoption, particularly when the proposed technology requires new test fixtures, material specifications, or changes to existing homologation procedures.

Safety design must also account for performance, comfort, and cost. Protective changes can alter mass distribution, cockpit packaging, visibility, and driver mobility, and poorly integrated systems can add ergonomic burdens even when they improve injury metrics [29,44]. This point is especially relevant to seats, restraint systems, and protective apparel, where fit and support affect both safety and driver function [46,65]. Therefore, the trade-off should not be understood simply as safety versus performance. Better fit, more stable restraint integration, and improved load distribution can support both protection and driver effectiveness. The central task is optimization under practical constraints rather than choosing between safety and competitiveness.

### 4.2. Future Directions for Motorsport Safety

The literature points most consistently to the need for stronger interdisciplinary collaboration. Biomechanical evidence is most useful when it informs engineering design, and material developments matter most when they are evaluated against injury mechanisms rather than structural metrics alone [13,35,56]. Shared validation datasets, more transparent model-development workflows, and closer collaboration among engineers, biomechanics, clinicians, and regulatory stakeholders would improve both reproducibility and translation [29,44,59].

Another clear direction is more individualized protection. Differences in body size, posture, and sex-related anthropometry can influence both biomechanical loading and the effectiveness of helmets, seats, suits, and restraint systems [28,46,66]. The available evidence therefore supports a gradual move away from one-size-fits-all protection toward driver-centered design. Real-time monitoring is also becoming more relevant. Driver-coupled sensing systems, especially instrumented mouthpieces and related head-kinematics tools, are improving the ability to capture race-environment loading rather than relying only on chassis-based or surrogate measurements [7,16]. When paired with telemetry, crash recorders, and video reconstruction, these data can improve injury surveillance, crash interpretation, and iterative refinement of helmets, restraints, and cockpit protection [8,12,44]. Broader implementation, however, will require larger datasets and more standardized collection practices.

### 4.3. Limitations

Several limitations should be acknowledged. First, the included studies were methodologically heterogeneous, which limits direct comparison across epidemiological, experimental, computational, and engineering evidence. Second, the review focused primarily on driver-centered protective systems and did not address vehicle-level crashworthiness in equal depth. Third, the literature remains weighted toward professional or better-instrumented settings, which may limit direct transferability to grassroots motorsport. Finally, as a scoping review, this study did not include a formal risk-of-bias assessment or quantitative meta-analysis. The qualitative classification of evidence maturity and primary study focus was based on the information reported in the included studies and should not be interpreted as a formal ranking of study quality or a numerical Technology Readiness Level assessment.

## 5. Conclusions

This scoping review indicates that motorsport safety is best advanced when biomechanics, protective-system design, and material selection are interpreted together rather than in isolation. Current evidence most clearly supports integrated head-neck protection, seat-restraint geometry, and the growing use of FEA to examine local injury mechanisms. The main gaps are sparse real-world surveillance, uneven validation of simulation-led findings, and limited driver-level evidence for many emerging material concepts. Future progress will depend on stronger surveillance, better motorsport-specific validation, and more driver-centered protective design.

## Figures and Tables

**Table 1:** Complete database-specific search strategies, search limits, and records identified.

Database	Complete Search Strategy	Search Limits and Search Date	Records Identified
PubMed	(((((motorsport [Title/Abstract]) OR (automotive racing [Title/Abstract])) OR (auto racing [Title/Abstract])) OR (racecar [Title/Abstract])) OR (competitive motor vehicle racing [Title/Abstract])) OR (high-performance vehicle racing [Title/Abstract])	Searches were limited to English-language records published between 2000 and July 2026; searched in July 2026	102
Web of Science	(((((AB = (motorsport)) OR AB = (automotive racing)) OR AB = (auto racing)) OR AB = (racecar)) OR AB = (competitive motor vehicle racing)) OR AB = (high-performance vehicle racing)	Searches were limited to English-language records published between 2000 and July 2026; searched in July 2026	681
IEEE Xplore	motorsport OR automotive racing OR auto racing OR racecar OR competitive motor vehicle racing OR high-performance vehicle racing	Searches were limited to English-language records published between 2000 and July 2026; searched in July 2026	186
Scopus	TITLE-ABS-KEY (motorsport) OR TITLE-ABS-KEY (automotive racing) OR TITLE-ABS-KEY (auto racing) OR TITLE-ABS-KEY (racecar) OR TITLE-ABS-KEY (competitive motor vehicle racing) OR TITLE-ABS-KEY (high-performance vehicle racing)	Searches were limited to English-language records published between 2000 and July 2026; searched in July 2026	681

Note: The publication-date limits were January 2000 through July 2026, and all databases were searched in July 2026. The exact day of each search was not recorded. The numbers of records identified represent the records retrieved before manual duplicate removal and sum to 1650.

**Table 2:** Summary of the primary evidence domains and characteristics of the 56 included studies.

Evidence Domain	Studies, *n* (%)	Study Designs	Context	Main Focus	Loading/Test	Outcomes	Evidence Contribution	Representative References
Injury mechanisms and biomechanical evidence	16 (28.6%)	Observational cohort, retrospective database, biomechanical experiment, epidemiological survey, injury reference review	Dirt track, NASCAR, IndyCar, WEC, rally, general automotive	Head, neck, spine, thoracolumbar, pelvis, lower extremity, brain	Real-world crash, rear impact, frontal impact, side impact, vertical impact	Head kinematics, injury risk, AIS, fracture, pain, IARV	Describes injury mechanisms, exposure, and risk in motorsport	[3,7,9,12,16,17,18,19,20,21,22,23,24,25,26,27]
Finite-element analysis and computational modeling	12 (21.4%)	FE model development/validation, parametric simulation, multibody + FE, optimization	NASCAR, F1, WEC, general road crash, track barrier	Hip, neck, thorax, spine, pelvis, arm	Frontal, side, rear, vertical impact, crash pulse	Stress/strain, injury metrics, kinematics, risk curves	Provides transferable injury prediction and parametric analysis tools	[28,29,30,31,32,33,34,35,36,37,38,39]
Protective-system design	14 (25.0%)	Sled test, drop test, component test, specification development, numerical simulation, interview/stakeholder study	NASCAR, F1, CART, FSAE, grassroots dirt, rally	Head/neck restraint, seat, seat belt, toe board, helmet, racing suit, medical rescue	Frontal, side, rear, vertical impact, HNR test	Force, acceleration, displacement, injury risk, test repeatability	Evaluates and optimizes restraints, seats, helmets, rescue, and standards	[8,40,41,42,43,44,45,46,47,48,49,50,51,52]
Material selection and impact mitigation	14 (25.0%)	Material characterization, quasi-static/dynamic compression, three-point bending, drop tower, FE material modeling, 3D printing	Race car energy absorber, helmet liner, track barrier, body structure	Energy-absorbing structure, sandwich, honeycomb, foam, composite, metamaterial	Axial crushing, bending, impact, thermoforming	SEA, peak force, CFE, modulus, damage morphology, manufacturability	Provides evidence for material selection and geometry design of energy-absorbing structures	[13,15,53,54,55,56,57,58,59,60,61,62,63,64]

NOTE: Data are presented as the number of included studies and the percentage of the total evidence base (*n* = 56). Each study was assigned to one primary evidence domain according to its main objective and methodological contribution; therefore, the domain totals sum to 56 and the percentages sum to 100%. The motorsport or adjacent context, anatomical focus, loading/test focus, and outcome measures summarize the predominant characteristics of each domain. The numbers in this column refer to the corresponding references in the manuscript reference list; the detailed study-level information is provided in Supplementary Table S1. FEA, finite-element analysis; WEC, World Endurance Championship; HNR, head-and-neck restraint; IARV, injury assessment reference value; SEA, specific energy absorption; CFE, crush force efficiency.

**Table 3:** Quantitative characterization of the 56 included studies by study design, publication period, and primary biomechanical or protection focus.

Classification Variable	Category	Number of Studies, *n* (%)
Study design	Observational, epidemiological, or field-based study	9 (16.1%)
	Biomechanical experiment, sled test, or prototype evaluation	11 (19.6%)
	Finite-element, computational, or numerical modeling	23 (41.1%)
	Materials characterization or component testing	9 (16.1%)
	Design, qualitative, review, or experience-based study	4 (7.1%)
Publication period	2000–2009	13 (23.2%)
	2010–2019	21 (37.5%)
	2020–2026	22 (39.3%)
Primary biomechanical or protection focus	Head or brain protection/kinematics	9 (16.1%)
	Neck or cervical biomechanics	11 (19.6%)
	Thorax or thoracolumbar spine	5 (8.9%)
	Pelvis or lower-extremity biomechanics	5 (8.9%)
	Integrated protective systems or driver protection	12 (21.4%)
	Materials and energy-absorbing structures	14 (25.0%)

**Note:** Each study was assigned to one primary category for each classification variable. For studies involving multiple methods or anatomical regions, classification was based on the dominant study objective and principal outcome reported in the source. Percentages are calculated using the total of 56 included studies and may not sum to 100% because of rounding.

**Table 4:** Representative injury characteristics and biomechanical indicators reported in motorsport crash studies.

Parameter	Observed/Reported Values	Injury Characteristics	References
Common Injuries	Concussion, Head/Neck Soreness, Fractures (knee, shoulder, thorax/rib, ankle), Sprains, Contusions, Abrasions	Most common injuries included the knee (12%), shoulder (11%), thorax/rib or ankle (9%), hand (8%), forearm (7%), and neck or wrist (5%)	[3,24]
Concussion Incidence	Contextual epidemiological values cited in motorsport head-kinematics literature: 1.3 concussions per 1000 professional saloon-car driver-races; 31% of surveyed drivers reported prior concussion	The occurrence rate of this event is higher than football (0.67/1000 exposures); 31% of drivers reported concussion in survey	[16]
Head Peak Linear Acceleration (PLA)	Crash events: median 12.3 g (95th percentile 37.3 g) in Zoch et al.; lap PLA: mean 5.57 g in Filben et al.; maximum observed: 26.8 g during perturbations and 36.6 g during crashes in Filben et al.	Head loading increases during crash events, and a single crash may involve multiple impacts	[44]
Head Peak Rotational Acceleration (PRA)	Median: 626 rad/s^2^ (95th percentile: 1799 rad/s^2^); Mean in normal laps: 198 rad/s^2^	Rotational acceleration is strongly implicated in concussion-related loading	[7]
Head Peak Rotational Velocity (PRV)	Median: 8.92 rad/s (95th percentile: 18.6 rad/s); Mean in normal laps: 3.07 rad/s	Higher rotational velocity is associated with more severe head-loading conditions	[7,44]
Vehicle Chassis g-Forces	Mean g for head injury: 79.6 (standard deviation: 28.5); no injury: 50.6 (standard deviation: 28.0); 50 g threshold: 16% head injury risk; 68 G: 10% probability; 149 g: 50% probability	g-forces on chassis correlate with head injury risk; ≥50 g is a key threshold	[26]
Directional Effects	Non-horizontal (rollover, vertical loads) and side impacts have greater head kinematics	Rollover and side impacts more likely to cause higher head loads	[7,44]
Injury Risk Models	The risk of injury increases with greater changes in velocity (ΔV), higher peak accelerations, and a higher number of impacts	Logistic regression models: AUROC ~0.80 for injury prediction using these variables	[12]
Protective Factors	Use of HANS, full containment seats, and energy-absorbing materials reduces severe injury risk	Professional series generally employ more comprehensive safety systems, whereas grassroots adoption and perceived effectiveness remain more variable	[24]

Note: “g” means acceleration due to gravity, a standard measure for impact forces. PLA means peak linear acceleration; PRA means peak rotational acceleration; PRV means peak rotational velocity; AUROC means area under the receiver operating characteristic curve.

**Table 5:** Summary of Driver Injuries by Crash Scenario and Evidence Type.

Crash Scenario/Direction	Primary Evidence Source/Design	Injury Location(s)	Injury Type/Severity	Biomechanical Mechanism	References
Frontal Impact	Computational/FE evidence	Thoracolumbar spine (T12–L2)	Compression fractures (Abbreviated Injury Scale (AIS) 2–3)	Axial compression & bending; risk reduced with increased seat ramp angle, slouched posture	[9,21,25]
Frontal Impact	Epidemiological/retrospective database evidence	Lumbar spine (L1, L2, L4, L5)	Burst/wedge compression fractures (AIS 3+)	Dynamic axial force through pelvis into seat pan; higher risk is associated with age, belted, bucket seats	[23]
Frontal Impact	Computational/FE evidence	Chest (ribs, sternum)	Rib fractures, sternum compression (AIS 1–3)	Direct impact with steering or belts; chest compression from restraint system	[29,35]
Frontal Impact	Computational/FE evidence	Head/neck	Concussion, mild traumatic brain injury, neck tension (AIS 1–2)	High deceleration, head/neck tension, helmet impact with steering or surround	[29,35]
Frontal Impact	Computational/FE evidence	Lower extremity (femur, tibia)	Femur/tibia fractures, ligament tears	Dashboard/knee impact, seat intrusion; fractures at 236 MPa, ligament tears at 70 MPa	[35]
Frontal Impact	Computational/FE evidence	Hip/acetabular region	Variation in acetabular injury tolerance/potential hip injury risk	Hip posture altered acetabular injury tolerance in frontal-collision FE simulations	[30]
Frontal Impact	Design study, questionnaire, and wear-test evidence	Fit-related discomfort/ergonomic concern	Potential fit-related disadvantage rather than a directly measured injury outcome	Unisex protective clothing may inadequately fit female drivers, especially at the shoulder, waist, hip, and crotch	[46]
Side Impact	Computational/FE evidence	Pelvis and thorax	Pelvic fractures and thoracic injury (AIS 3+)	Lateral loading, side intrusion, seat/door impact, bending/shear/burst loading	[34,36]
Rear Impact	Computational/FE, cadaveric, and experimental/prototype evidence	Cervical spine (neck, facet joints)	Soft tissue, facet capsule strain, neck shear (AIS 1–2)	Hyperextension, ramping, shear force at neck, gender/age effects	[22,31,42]
Oblique/Frontal-Oblique Impact	Computational/FE evidence	Thoracolumbar spine, chest	Fractures, chest compression	Combined axial/bending, seat angle, restraint geometry	[9,25]
Lateral Impact (Neck)	Computational/FE evidence	Cervical spine	Neck shear, coronal moment (AIS 3+)	Lateral shear force at occipital condyle/T1; 1.5 kN at 50% risk	[32]

Note: AIS refers to the Abbreviated Injury Scale, an anatomically based system for classifying injury severity. FE refers to finite-element analysis. The primary evidence source/design was assigned according to the study design of the cited references supporting each row. When multiple study designs contributed to a row, the evidence types were listed together. This classification describes the source of evidence and does not represent a formal ranking of evidence quality. T1 means the first thoracic vertebra; T12–L2 means thoracic vertebra 12 through lumbar vertebra 2; L1, L2, L4, and L5 mean lumbar vertebrae 1, 2, 4, and 5, respectively.

**Table 6:** The evolution of motorsport helmet design.

Period	Helmet Type & Design	Key Features & Advancements	Safety Impact & Findings	References
Early designs	Early helmet configurations	Basic shell protection with limited padding and no integrated head-neck restraint interface	Provided blunt-impact protection but limited control of head excursion and neck loading	[43,48]
Pre-HANS era	Full-face and certified helmet development	Expanded facial coverage, improved fit, and broader use of certified shell-liner systems	Improved frontal and debris protection, although head-neck loading remained a concern in severe crashes	[43,48]
1990s	Standardized, Certified Helmets (Snell/FIA)	Industry adoption of certification standards (Snell, FIA), improved materials (e.g., composites, energy-absorbing foam liners), integration with harness systems	Improved standardization and compatibility with increasingly sophisticated restraint systems	[17,28,40]
Early 2000s	Integration with HANS Devices	Helmets designed to interface with HANS devices (using tethers), improved fitment, focus on reducing head excursion and neck loads; development of helmet mounting points for HANS tethers.	Biomechanical studies indicate reduced upper-neck loading during severe frontal impacts. The present review does not quantify population-level injury reductions or establish regulatory causation.	[41,43,48]
2000s–2010s	Advanced Materials, Custom Fit, Enhanced Energy Absorption	Use of advanced composite shells (carbon fiber), improved viscoelastic and energy-absorbing foams, custom fit using 3D scans, better integration with seat and restraint systems; improved testing methods (e.g., FE models).	Potentially improved head and neck protection, although the reviewed studies primarily reported biomechanical or modeled outcomes.	[17,28,40,43,48]
2010s–Present	Enhanced Helmet Systems (EHS); Accessory Integration	Addition of external attachments (visors, cameras); helmets modeled and tested with add-ons in computational studies; focus on both linear and angular acceleration mitigation; further emphasis on gender/anthropometry fit.	Accessory additions such as visors and cameras appear to have relatively small effects on modeled injury metrics; helmet mass and fit remain important	[29]
Current and emerging directions	Sensor-enabled and simulation-informed helmet concepts	Wearable sensing, telemetry integration, and parametric evaluation across driver sizes and crash types	Potential for improved monitoring and more individualized helmet-restraint optimization; evidence remains developmental	[28,29,43,48]

**Table 7:** Evidence maturity and validation status of material classes identified in the review.

Material Class	Representative Safety Application	Evidence Maturity	Validation Reported in the Reviewed Studies	Evidence Maturity and Translation Status
CFRP and other composite structures	Energy-absorbing structures and safety-cell components	Relatively established engineering evidence	Component testing, crash testing, certification-oriented testing, or FE-supported evaluation	Formal TRL was not reported; evidence remains application- and component-dependent
Aluminum honeycomb and sandwich structures	Energy absorption and structural load management	Moderate engineering evidence	Material characterization, mechanical testing, crash modeling, or component testing	Formal TRL was not reported; integration and failure behavior require further validation
Polymeric foams and energy absorbers	Cockpit padding, toe-board, seat, and helmet-liner applications	Moderate, application-dependent evidence	Compression, drop, sled, or component testing	Formal TRL was not reported; durability and environmental effects remain relevant
Additively manufactured and topology-optimized structures	Lightweight cores and impact attenuators	Emerging evidence	Numerical optimization, simulation, and laboratory or component testing	Formal TRL was not reported; manufacturing repeatability and certification remain unresolved
Auxetic, cellular, metamaterial, and bio-inspired structures	Tunable impact absorption and helmet-liner concepts	Early-stage evidence	Mainly simulation or small-scale laboratory testing	Formal TRL was not reported; driver-level and field validation are limited
Natural-fiber and recycled composites	Lightweight structural and energy-absorbing components	Early-stage evidence	Material-level mechanical testing	Formal TRL was not reported; durability, variability, and certification require further study

Note: Evidence maturity was assessed qualitatively according to the type, scale, and extent of validation reported in the included studies. In Supplementary Table S1, the column “TRL reported by the study” records whether a formal Technology Readiness Level (TRL) was explicitly reported by the original study; “NR” indicates that no formal TRL was reported. Because formal TRLs were not consistently reported, the material-class summaries in this table represent review-level interpretations of application maturity and translation status rather than study-reported TRLs. They should not be interpreted as formal evidence-quality rankings, FIA homologation, regulatory approval, or demonstrated reduction in real-world driver injuries. The material classifications were derived from the study-level information provided in Supplementary Table S1.

## Data Availability

All data charted during this scoping review are provided in the main manuscript tables and Supplementary Table S1. The corresponding author can provide additional clarification regarding the exclusion decisions if required.

## Supplementary Materials

The following supporting information can be downloaded at: https://www.mdpi.com/article/10.3390/bioengineering13101159/s1, Table S1: Study-level data-charting table for the 56 included studies. Table S2: PRISMA-ScR Checklist [67].

## Abbreviations

The following abbreviations are used in this manuscript:

g	Acceleration due to gravity
AIS	Abbreviated Injury Scale
AUROC	Area under the receiver operating characteristic curve
CFRPs	Carbon-fiber-reinforced polymers
EHS	Enhanced Helmet Systems
FEM	Finite element modeling
FEA	Finite element analysis
HANS	Head and Neck Support
PLA	Peak linear acceleration
PRA	Peak rotational acceleration
PRV	Peak rotational velocity
ΔV	Change in velocity
T1	First thoracic vertebra
T12-L2	Thoracic vertebra 12 to lumbar vertebra 2
L1, L2, L4, L5	Lumbar vertebrae 1/2/4/5
